# Exploring the Association Between Parenting Styles, Dental Anxiety, and Dental Caries Experience Among Children Attending a University Dental Clinic: A Cross-Sectional Study

**DOI:** 10.3390/dj14100673

**Published:** 2026-10-15

**Authors:** Vivek Padmanabhan, Md Sofiqul Islam, Nada Tawfig Hashim, Nallan C. S. K. Chaitanya, Muhammed Mustahsen Rahman, Sara Mohammed Jakka Al Mansoori, Hebah Abdelrahman

**Affiliations:** RAK College of Dental Sciences, RAK Medical and Health Sciences University, Ras Al Khaimah 12973, United Arab Emirates; sofiqul.islam@rakmhsu.ac.ae (M.S.I.);

**Keywords:** dental caries, dental anxiety, parenting style, children, pediatric dentistry, mediation analysis

## Abstract

**Background:** Parenting style may influence children’s oral health behaviours and responses to dental care, but its joint relationship with dental anxiety and caries has rarely been examined. This study assessed these associations and whether dental anxiety mediated the association between parenting style and caries experience. **Methods:** In this cross-sectional study, 143 children aged 6–12 years attending a university pediatric dental clinic in the United Arab Emirates were assessed using a 30-item adaptation of the Parenting Styles and Dimensions Questionnaire, the Modified Dental Anxiety Scale, and the DMFT/dmft index. Mediation was tested in a single bootstrapped model containing all three parenting dimensions, age, and sex. **Results:** Parenting was classified as authoritative (*n* = 127), authoritarian (*n* = 4), or permissive (*n* = 12). Parenting style was associated with dental anxiety (*p* = 0.016) and caries experience (*p* < 0.001), and anxiety correlated with caries experience (ρ = 0.314, *p* < 0.001). Dental anxiety accounted for approximately 39% of the association between the authoritative dimension and caries experience (ACME = −0.260, *p* = 0.006), and this indirect effect remained significant in children aged 9–12 years. For the permissive dimension, only the direct effect was significant (*p* < 0.001). **Conclusions:** These exploratory findings, obtained with markedly imbalanced parenting-style groups, require confirmation in larger, more balanced, longitudinal samples.

## 1. Introduction

Dental caries in children remains among the most prevalent chronic oral health conditions globally; it continues to pose a definite and substantial public health burden despite remarkable advances in prevention and treatment strategies [1,2,3]. Dental caries is a biofilm-mediated condition with multifactorial aetiology, which occurs as an outcome of interactions among cariogenic microbes, saliva, fermentable carbohydrates, and host susceptibility [1,4]. In addition to well-established risk factors such as oral hygiene practices, dietary habits, socioeconomic status, and fluoride exposure, available evidence suggests that family-related and psychosocial factors may also influence children’s oral health behaviours, which may then affect their dental caries experience [5,6]. Dental anxiety has received considerable attention in pediatric dentistry among the psychosocial factors associated with oral health [7,8]. Dental anxiety is characterized by fear, stress, or apprehension associated with dental treatment and is one of the most common behavioural challenges encountered during the provision of dental care to children [7,9,10]. Higher dental anxiety has been linked to delayed or missed dental visits, reduced use of preventive dental services, and poorer cooperation during treatment [7,8,11]. Dental anxiety may contribute to avoidance or postponement of dental care, potentially allowing untreated disease to progress. Conversely, children with greater caries experience may require more extensive dental treatment, which may contribute to further dental fear or anxiety [7,8]. This reciprocal relationship highlights the importance of understanding the factors that contribute to anxiety in children receiving dental care.

Parenting style has been found to be one of several factors that may affect the development of children’s emotional and behavioural responses to dental care [12,13,14]. Parenting styles are defined as characteristic patterns of parental attitudes, expectations, and behaviours used in raising children and have a possible influence on emotional development, the development of coping abilities, and health-related behaviours in children [12,13]. Baumrind originally described three principal parenting styles: authoritative, authoritarian, and permissive [15]. Authoritative parenting provides appropriate behavioural control combined with warmth and responsiveness, which may foster self-confidence and independence in children. Authoritarian parents emphasize obedience, strict discipline, and high parental control with limited emotional responsiveness. Permissive parents are nurturing and accepting but set fewer rules or behavioural expectations [15]. These different parenting approaches may influence children’s oral hygiene practices, dietary behaviours, adherence to preventive dental care, and emotional responses to dental treatment [16,17,18]. As a result, parenting style may affect dental caries both directly through oral hygiene practices and through pathways involving dental anxiety [13,16,19].

Several mechanisms have been proposed to connect parenting style with children’s oral health. The first is behavioural regulation: the establishment and maintenance of twice-daily toothbrushing, the regulation of between-meal sugar exposure, and adherence to scheduled preventive appointments all depend on parental structure, supervision, and consistent follow-through, and each of these is an established determinant of caries incidence. The second is modelling, whereby children acquire oral health practices by observing and imitating the behaviour of their parents. The third is emotional socialization: the way in which parents respond to a child’s distress shapes the coping strategies that the child brings to unfamiliar or threatening situations, of which dental treatment is a common example in childhood. Authoritative parenting, which combines high responsiveness with age-appropriate demands, has been associated with better emotional regulation and more adaptive coping, whereas permissive parenting, characterized by high responsiveness but few consistent demands, provides fewer opportunities for children to develop these strategies [12,13].

These mechanisms imply that parenting style may influence dental caries experience along more than one route: a behavioural route operating through oral hygiene and dietary practices, and a psychological route operating through the child’s emotional response to dental care and consequent patterns of attendance and cooperation. Distinguishing between these routes requires an analytical framework capable of separating them. Mediation analysis decomposes the total association between an exposure and an outcome into an indirect component transmitted through an intermediate variable and a direct component that operates through all remaining pathways. Quantifying the proportion of the association that is transmitted through dental anxiety is of practical interest, because anxiety is modifiable through established behaviour-management techniques, whereas parenting style is not readily altered within a dental setting.

Previous research has independently demonstrated associations between dental anxiety and dental caries, and between parenting style and children’s oral health behaviours [8,16,20]. Recent evidence also indicates that parenting style may influence children’s behaviour and emotional responses in dental settings [14,18]. However, there is a paucity of studies that investigate the association between these three variables simultaneously, and to the authors’ knowledge these interrelationships have not been examined collectively among children in the United Arab Emirates, a population with a high reported burden of childhood caries and a culturally heterogeneous resident community. The current cross-sectional study was therefore designed with three objectives: first, to describe the association between parenting style and dental anxiety; second, to examine the association between parenting style and dental caries experience, and between dental anxiety and dental caries experience; and third, to explore, using mediation analysis, whether dental anxiety accounts for part of the association between parenting style and dental caries experience. Consistent with the mechanisms outlined above, it was anticipated that the authoritative dimension would be associated with lower dental anxiety and lower caries experience and that a portion of this association would be transmitted through dental anxiety. Given the cross-sectional design, all analyses were regarded as exploratory and hypothesis-generating rather than confirmatory.

## 2. Materials and Methods

### 2.1. Study Design, Setting, and Participants

The current study, with a cross-sectional design, included children attending the university dental clinics of RAK College of Dental Sciences, RAK Medical and Health Sciences University, United Arab Emirates. Institutional approval was obtained from RAK Medical and Health Sciences University on 19 November 2024 (RAKMHSU-HEC-107-2024/25-F-D). Because the study involved children, external ethical review was additionally required, and approval was obtained from the Ministry of Health and Prevention Research Ethics Committee, RAK Subcommittee, on 6 February 2025 (MOHAP/REC/2025/7-2025-UG-D). The study adhered to the ethical principles outlined in the Declaration of Helsinki, and written informed parental consent and child assent were obtained before any study procedure. Participant enrolment and data collection were carried out between February 2025 and July 2025, after both approvals had been granted. Systemically healthy children aged 6–12 years attending during the recruitment period were recruited consecutively. Children with developmental disorders, systemic illnesses, or children who were on long-term medications that could influence oral health, general health, or anxiety were excluded from the study.

During the study period, 182 children aged 6–12 years attending the pediatric dental clinics were assessed for eligibility. Thirty-two children or their parents declined to participate. Four children were excluded because of long-term medication that could influence oral health, general health, or anxiety, and three were excluded because the parent questionnaire was returned incomplete. The final analytical sample therefore comprised 143 children with complete data on all study variables. All study instruments were administered in English.

The university teaching dental hospital provides dental care to residents of Ras Al Khaimah and the surrounding Northern Emirates. The pediatric dentistry clinics receive both directly attending patients and internal referrals, and children present for a range of reasons including routine examination, preventive care, and symptomatic complaints. On each clinic session, children within the eligible age range were identified from the appointment list and were approached together with their accompanying parent or legal guardian, and the purpose and procedures of the study were explained before consent was sought. 

### 2.2. Assessment of Parenting Style

Parenting style was assessed using a 30-item adaptation of the Parenting Styles and Dimensions Questionnaire (PSDQ) [21], self-administered by the parent. The instrument comprised 13 items assessing the authoritative dimension, 13 assessing the authoritarian dimension, and 4 assessing the permissive dimension. Items were rated on a six-point response format anchored by “never” (1) and “always” (6), and a mean score was calculated for each dimension by dividing the subscale total by its number of items. This response format differs from the five-point format of the original PSDQ, and dimension scores are therefore not directly comparable with published PSDQ norms; to allow comparison with published values, scores were additionally expressed on an equivalent five-point metric (Section 2.6). Internal consistency in the present sample was good for all three dimensions (Cronbach’s α = 0.84 authoritative, 0.83 authoritarian, 0.81 permissive).

The PSDQ scoring protocol yields three continuous dimension scores rather than a categorical classification. Continuous dimension scores were therefore used in the primary analyses. For descriptive and secondary categorical analyses, each parent was additionally classified according to the dimension with the highest mean score, and the margin between the highest and second-highest dimension score was recorded to indicate how clearly each parent was classified. Because the instrument does not assess an uninvolved or neglectful dimension, that style was not evaluated.

The questionnaire was completed by the parent or legal guardian accompanying the child, in a quiet area of the clinic and before the child’s clinical examination. Where more than one caregiver attended, the questionnaire was completed by the primary caregiver. Parents completed the instrument independently; investigators were available to clarify procedural queries but provided no guidance on the content of individual items or on the response that should be selected. Completed questionnaires were checked for missing responses at the point of collection, and children whose questionnaires were returned incomplete were excluded at enrolment rather than analyzed with missing data (Section 2.1). 

This adapted 30-item, six-point version has not undergone formal population-specific validation in the United Arab Emirates; this limitation is considered in the interpretation of the findings.

### 2.3. Assessment of Dental Anxiety

A five-item Modified Dental Anxiety Scale (MDAS) was used for evaluating dental anxiety, with each item scored from 1 to 5 and total scores ranging from 5 to 25 [22]. For categorical analysis, MDAS scores were classified as low dental anxiety (5–9), moderate dental anxiety (10–18), and high dental anxiety (19–25) [23]. Children completed the scale independently whenever possible. Where a younger child requested clarification, the investigator provided verbal examples to aid comprehension without suggesting or influencing a response; parents were permitted to interpret items but not to answer on the child’s behalf. The examples used were not scripted, and this is acknowledged as a potential source of measurement variability. Recent evidence supports the reliability and validity of the MDAS in children aged 9–12 years [24]; because the present sample also included children aged 6–8 years, for whom the instrument has not been validated, MDAS scores were compared between the 6–8 and 9–12 age groups and the principal analyses were repeated in the 9–12 subgroup alone (Section 2.6).

### 2.4. Assessment of Dental Caries Experience

Dental caries experience was assessed in accordance with the criteria provided by the World Health Organization [25], using the DMFT index for permanent teeth and the dmft index for primary teeth. For children with mixed dentition, the combined DMFT/dmft score represented the sum of affected permanent and primary teeth. Two calibrated examiners performed clinical assessments. Before data collection, they completed standardization and calibration exercises in children who were not included in the study. Inter-examiner agreement for DMFT/dmft recording was almost perfect (Cohen’s κ = 0.82). The study was reported following the Strengthening the Reporting of Observational Studies in Epidemiology (STROBE) guidelines [26].

Examinations were conducted in the dental clinic with the child seated in a dental chair under standard operatory illumination, using a plane mouth mirror and a ball-ended Community Periodontal Index (CPI) probe (Himmel Tek, Sharjah, United Arab Emirates), with the tooth surfaces dried before assessment. Radiographs were not obtained for the purposes of the study; therefore, approximal lesions not detectable by visual and tactile examination would not have been recorded. This approach is consistent with the World Health Organization basic methods protocol but may result in a conservative estimate of caries experience. Each child was examined once by a single examiner, with the questionnaire data unavailable to the examiner at the time of examination. 

### 2.5. Sample Size and Power

The sample size was estimated a priori for the primary continuous-variable analysis of the association between dental caries and dental anxiety. With a two-sided α of 0.05, 80% power, and an anticipated correlation of 0.30, at least 84 participants were required [27]. The final sample of 143 exceeded this requirement. The sample size was not determined with reference to the expected distribution of parenting styles, and the study was therefore not powered for comparisons between parenting-style subgroups.

### 2.6. Statistical Analysis

Data analysis was performed using IBM SPSS Statistics for Windows, version 29.0 (IBM Corp., Armonk, NY, USA) and R version 4.6.1 (R Foundation for Statistical Computing, Vienna, Austria). The distribution of continuous variables was examined for normality using the Shapiro–Wilk test together with visual inspection of Q–Q plots. Participant characteristics were summarized using descriptive statistics. Numerical data were reported as mean with standard deviation or median with interquartile range, depending on the distribution, and categorical data as counts with percentages. Clinical findings and questionnaire responses were recorded on paper proformas and subsequently entered into Microsoft Excel for Mac (Microsoft 365; Microsoft Corporation, Redmond, WA, USA) by one investigator, with entries verified against the source records by a second investigator. Because children with incomplete questionnaires were excluded at enrolment, the analytical dataset of 143 children was complete for every study variable, and no imputation of missing values was required.

The Shapiro–Wilk test indicated that the continuous variables were not normally distributed; therefore, non-parametric statistical methods were employed. The association between dental anxiety and parenting style was evaluated using Fisher’s exact test because several expected cell counts were less than five. The Kruskal–Wallis test, followed by Dunn’s post hoc test with Benjamini–Hochberg adjustment for pairwise comparisons, was used to examine DMFT/dmft scores among parenting styles. Spearman’s rank correlation coefficient was used to assess the association between dental anxiety (MDAS score) and dental caries experience (DMFT/dmft). Associations of age and sex with the study variables were examined using Spearman’s rank correlation and the Mann–Whitney U test, respectively.

Continuous PSDQ dimension scores were used in the primary mediation analyses. Because the three dimensions were measured in the same respondents and may covary, a single multivariable model was specified rather than separate models for each dimension. All three dimension scores, together with the child’s age and sex, were entered as covariates in both the mediator model (MDAS score regressed on the three dimensions, age, and sex) and the outcome model (DMFT/dmft regressed on the three dimensions, MDAS score, age, and sex). The average causal mediation effect (ACME) represented the indirect effect through dental anxiety, the average direct effect (ADE) represented the effect not operating through dental anxiety, and the total effect represented the overall effect. Effects were estimated for each dimension from this joint model using the latest version of the mediation package in R [28], with nonparametric bootstrapping and 5000 simulations. Multicollinearity among the three dimension scores was assessed using variance inflation factors, which were 2.29, 1.23, and 2.19 for the authoritative, authoritarian, and permissive dimensions, respectively.

Two sensitivity analyses were performed. First, the three dimensions were modelled separately, without mutual adjustment, to permit comparison with the conventional single-exposure specification. Second, the joint model was refitted in children aged 9–12 years only, the age range for which the MDAS has been validated. In addition, to allow comparison with published PSDQ values, dimension scores were linearly rescaled from the six-point to an equivalent five-point metric, and the joint model was refitted. Because this transformation is linear and applied identically to every participant, it changes the scale of the coefficients but cannot alter statistical inference or parenting-style classification; it is therefore reported for comparability and not as a test of robustness.

Because the sample size was determined a priori for the correlation between dental anxiety and dental caries experience, the study was not powered to compare indirect effects between parenting dimensions, and no such comparison was undertaken. A non-significant indirect effect indicates that no statistically significant mediation was detected in these data and should not be interpreted as evidence that mediation is absent. Given the cross-sectional design, the mediation analysis was considered exploratory and was not intended to establish causal mediation: temporal ordering between dental anxiety and dental caries experience could not be established, and unmeasured confounding of the exposure–mediator, mediator–outcome, and exposure–outcome pathways could not be excluded. A two-sided *p* < 0.05 was considered statistically significant.

## 3. Results

### 3.1. Participant Characteristics and Parenting-Style Scores

A total of 143 children aged 6–12 years participated in the study, with a mean age of 8.92 ± 1.70 years; 59 children (41.3%) were aged 6–8 years and 84 (58.7%) were aged 9–12 years. Of the participants, 71 (49.7%) were male and 72 (50.3%) were female. Overall, 119 children (83.2%) had a DMFT/dmft score greater than zero, while 24 (16.8%) had a score of zero. Descriptive characteristics, PSDQ dimension scores, and the distribution of parenting styles and dental anxiety levels are presented in Table 1.

The authoritative dimension recorded the highest mean score (5.15 ± 1.08), followed by the permissive (2.54 ± 0.96) and authoritarian (2.37 ± 0.75) dimensions. The margin between each parent’s highest and second-highest dimension score had a median of 3.00 (Q1–Q3 2.60–3.30) with a minimum of 1.90, and no parent had a margin below 1.00; classification into a dominant parenting style was therefore unambiguous for every participant. Based on the highest dimension score, authoritative parenting was the dominant style (88.8%), followed by permissive (8.4%) and authoritarian (2.8%). Low dental anxiety was observed in 40.6% of participants, moderate anxiety in 39.2%, and high anxiety in 20.3%.

### 3.2. Age and Sex

Age was not associated with dental caries experience (Spearman’s ρ = −0.067, *p* = 0.424) or with dental anxiety (ρ = −0.090, *p* = 0.284) and did not differ across parenting-style groups (Kruskal–Wallis *p* = 0.301). Caries experience did not differ by sex (*p* = 0.322). MDAS scores were somewhat higher in boys (12.99 ± 5.47) than in girls (11.31 ± 5.55), but the difference did not reach statistical significance (*p* = 0.058). Age and sex were nevertheless retained as covariates in the multivariable mediation models.

### 3.3. Parenting Style, Dental Anxiety, and Caries Experience

Parenting style was significantly related to dental anxiety (Fisher’s exact test, *p* = 0.016), with children exposed to permissive parenting showing the highest proportion of high dental anxiety (58.3%), whereas those raised with authoritative parenting more commonly reported low to moderate anxiety. However, because only 4 children were classified as having authoritarian and 12 as having permissive parenting, any comparison involving these groups is based on very few observations and should be interpreted with caution (Table 2).

A statistically significant difference in dental caries experience (DMFT/dmft) was observed across the three parenting styles (Kruskal–Wallis test, H = 32.80, *p* < 0.001). Dunn’s post hoc test with Benjamini–Hochberg adjustment showed that children with permissive parenting had significantly higher DMFT/dmft scores than those with authoritative parenting (adjusted *p* < 0.001) and authoritarian parenting (adjusted *p* = 0.039). However, no statistically significant difference was observed between the authoritative and authoritarian parenting groups (adjusted *p* = 0.393). The authoritative–permissive contrast involves 127 and 12 children, respectively, and is the only comparison with appreciable statistical power; both comparisons involving the authoritarian group rest on four observations (Table 3, Figure 1).

Furthermore, the correlation analysis showed a statistically significant positive correlation between dental anxiety and dental caries experience (Spearman’s ρ = 0.314, *p* < 0.001): the higher the MDAS scores, the higher the DMFT/dmft scores (Figure 2). The strength of this association was weak to moderate, indicating a modest positive relationship between dental anxiety and dental caries experience.

### 3.4. Dental Anxiety by Age Group

MDAS scores did not differ significantly between children aged 6–8 years (12.64 ± 5.73, *n* = 59) and those aged 9–12 years (11.79 ± 5.44, *n* = 84; Mann–Whitney U, *p* = 0.373), nor did caries experience (4.39 ± 3.67 versus 3.62 ± 2.63, *p* = 0.313). The correlation between MDAS score and DMFT/dmft was, however, stronger in the younger group (ρ = 0.449, *p* < 0.001) than in the older group (ρ = 0.174, *p* = 0.114); the difference between these coefficients did not reach statistical significance (Fisher’s *z* = 1.77, *p* = 0.076). This pattern is examined further in the sensitivity analyses.

### 3.5. Mediation Analysis

Mediation analysis was performed to examine whether dental anxiety explained part of the relationship between parenting style and dental caries experience (Table 4). In the multivariable model containing all three PSDQ dimensions together with age and sex, a statistically significant indirect effect through dental anxiety was observed for the authoritative dimension (ACME = −0.260, 95% CI −0.526 to −0.072, *p* = 0.006), corresponding to approximately 39% of its total effect (−0.667, *p* = 0.032); the direct effect for this dimension was not statistically significant (ADE = −0.407, *p* = 0.183). For the permissive dimension, the indirect effect through dental anxiety was not statistically significant (ACME = −0.122, *p* = 0.192), whereas the direct effect (ADE = 1.497, *p* < 0.001) and total effect (1.376, *p* < 0.001) were both significant. No statistically significant effects were observed for the authoritarian dimension. These results indicate that the association between the authoritative dimension and lower caries experience was partly accounted for by lower dental anxiety, whereas for the permissive dimension no statistically significant indirect effect through dental anxiety was detected, while the direct association with caries experience remained statistically significant. This suggests that pathways other than dental anxiety may contribute to the observed association, although a role for dental anxiety cannot be excluded.

### 3.6. Sensitivity Analyses

The indirect effect for the authoritative dimension remained statistically significant when the three dimensions were modelled separately without mutual adjustment and when the analysis was restricted to children aged 9–12 years (Table 5). For the permissive dimension, the indirect effect was positive in the separate model (0.109) but negative in the joint model (−0.122). This reversal after mutual adjustment is consistent with the shared variance between the authoritative and permissive dimensions and indicates that the permissive indirect estimate is sensitive to model specification; its direction is therefore not interpreted. In the rescaled joint model, coefficients changed by the constant factor of the rescaling (5/4), as expected by construction; small differences in *p*-values reflect bootstrap simulation variability. In the 9–12 subgroup, indirect effects for the authoritarian and permissive dimensions also reached nominal significance, both with negative estimates; however, the corresponding total effects were not significant, and the subgroup comprises 84 children, so these estimates are unstable and are not interpreted further.

For reference, when expressed on the equivalent five-point metric the dimension means were 4.32 ± 0.87 for the authoritative, 2.09 ± 0.60 for the authoritarian, and 2.23 ± 0.77 for the permissive dimension.

## 4. Discussion

The current cross-sectional research examined the relationships between parenting style, dental caries burden, and dental anxiety in children attending a university pediatric dental clinic in the United Arab Emirates. Parenting style was associated with dental anxiety and with caries experience, and dental anxiety itself correlated positively with caries experience. In a multivariable mediation model, the association between the authoritative dimension and lower caries experience was partly accounted for by lower dental anxiety, whereas for the permissive dimension no statistically significant indirect effect through dental anxiety was detected, although its direct association with higher caries experience remained significant.

The observed association between parenting style and dental anxiety is consistent with previous research suggesting that parenting practices may influence children’s behavioural adaptation and emotional response during dental care, with authoritative parenting generally associated with more adaptive behaviour and permissive parenting with less favourable behavioural responses [13,14,18,29,30]. These findings may be explained by the salient features of authoritative parenting, which combines emotional warmth with appropriate behavioural guidance and promotes emotional regulation and adaptive coping strategies during stressful situations. In contrast, permissive parenting is characterized by fewer behavioural boundaries and expectations, which may restrict the development of effective coping mechanisms when faced with unfamiliar dental procedures, therefore increasing susceptibility to dental anxiety [13].

Children exposed to permissive parenting exhibited higher DMFT/dmft scores when compared with children raised with authoritative parenting. Systematic reviews and other studies have reported that authoritative parenting is generally related to better oral hygiene practices, healthier dietary habits, greater parental supervision, and more regular utilization of preventive dental services, whereas permissive parenting has been associated with poorer oral health behaviours and a greater risk of dental caries [16,17,29,31]. However, the wider evidence base is more equivocal than the present findings alone would suggest, and the difference merits closer examination. In a cross-sectional study of 210 parent–child dyads that adjusted for dietary intake, socioeconomic position, parental education, and payment type using multivariable regression, parenting style showed no independent association with dmft, whereas parental education did [32]. A 2024 systematic review of nine studies likewise found the evidence divided: three studies reported a significant association between parenting style and caries experience, four found no correlation, and two found no significant difference between styles [19].

Several features of the present study may account for the stronger association observed here. First, the sample was recruited from a university dental clinic and therefore comprises treatment-seeking children, among whom caries experience and dental anxiety are both likely to be higher than in the general pediatric population; 83.2% of participants had a DMFT/dmft score greater than zero. Second, the permissive group comprised only 12 children with a narrow and uniformly high range of caries scores, so the magnitude of the group difference is estimated with considerable uncertainty. Third, and in contrast to the study by Ayoub et al. [32], dietary, socioeconomic, and oral-hygiene information was not collected in the present study, and the association between parenting style and caries experience is therefore unadjusted for these factors; the observed difference may partly reflect confounding by them. Fourth, the parenting instrument used here was a 30-item adaptation administered on a six-point response format, and dimension scores are consequently not directly comparable with those of studies using the standard version. When expressed on an equivalent five-point metric, the mean authoritative score in this sample (4.32) was similar to the value reported in a recent validation of the short-form PSDQ among parents attending dental clinics (4.35) [33], and internal consistency was comparable for the authoritative dimension; however, similar mean scores do not establish equivalent measurement properties, and the adapted instrument has not been formally validated in this population.

Within the sample of the present study, dental anxiety correlated positively with caries experience, wherein greater anxiety was related to higher DMFT/dmft scores. This observation agrees with earlier research reporting that greater dental anxiety is related to less favourable oral health outcomes in children and adolescents [7,8,20]. Because the design was cross-sectional, this correlation is compatible with the bidirectional relationship outlined in the Introduction and does not indicate which of the two precedes the other. This correlation was stronger among children aged 6–8 years than among those aged 9–12 years, although the two coefficients did not differ significantly. Because the MDAS has been validated in children from the age of nine, and younger children were more likely to require verbal clarification of individual items, the possibility of differential measurement error in the younger group cannot be excluded. Reassuringly, the indirect effect for the authoritative dimension remained statistically significant when the analysis was restricted to children aged 9–12 years.

The mediation analysis provided further insight into these relationships. Approximately 39% of the total association between the authoritative dimension and caries experience operated through dental anxiety, with the remaining direct effect no longer statistically significant after mutual adjustment. This is consistent with a pathway in which warmth combined with consistent behavioural guidance supports emotional regulation, lowers anxiety in the dental setting, and thereby facilitates acceptance of care. For the permissive dimension, no statistically significant indirect effect through dental anxiety was detected, whereas the direct association with caries experience remained statistically significant. This suggests that pathways other than dental anxiety—such as reduced supervision of toothbrushing, less consistent limit-setting around cariogenic foods, and irregular preventive attendance—may contribute to the observed association, although a role for dental anxiety cannot be excluded given the limited precision of the estimate. The permissive indirect estimate also changed sign between the separate and joint models, so its direction should not be interpreted. Although mediation studies in pediatric dentistry are limited, previous research has highlighted the potential contribution of psychological and family-related factors to children’s oral health behaviours and dental service utilization [34] and interrelationships among parental characteristics, child psychological and behavioural factors, and dental attendance have been examined using path-analysis and conceptual-modelling approaches [35,36]. Because the present study was cross-sectional, the temporal ordering of parenting style, dental anxiety, and caries experience cannot be established, and the mediation findings should be interpreted as a statistical decomposition of the observed associations that is consistent with an indirect pathway rather than as evidence of a causal mediating mechanism.

### 4.1. Limitations

Several limitations should be considered when interpreting these results. The cross-sectional design precludes conclusions regarding causality or temporal ordering among parenting style, dental anxiety, and dental caries experience, including causal interpretation of the mediation analysis. The study was conducted at a single centre in a treatment-seeking population, which limits generalizability. The distribution of parenting styles was markedly imbalanced, with 88.8% of parents classified as authoritative and only 4 and 12 classified as authoritarian and permissive respectively; comparisons involving these subgroups are consequently imprecise, and the study was not powered for them. Future work should be designed to yield more comparable subgroup sizes—for example, by screening on parenting style before enrolment or by purposively oversampling non-authoritative parents—so that the role of dental anxiety in the relationship between parenting style and caries experience can be examined more reliably.

The parenting instrument was a 30-item adaptation of the PSDQ administered on a six-point rather than the original five-point response format. Although internal consistency was good, dimension scores are not directly comparable with published PSDQ norms, and the adapted version has not itself been formally validated in this population. Parent-reported measures of parenting are additionally susceptible to social desirability and acquiescence bias, which may contribute to the high proportion of parents classified as authoritative.

The MDAS has been validated in children aged 9–12 years but not in younger children, and verbal examples were provided to some younger participants without a standardized script, which may have introduced measurement variability. The study did not collect detailed dietary, socioeconomic, or oral-hygiene information, so confounding by factors such as parental education, socioeconomic status, dietary practices, and oral-hygiene behaviours could not be evaluated; age and sex were adjusted for in the multivariable models, but residual confounding is likely. Consequently, the observed associations between parenting style and dental caries experience should not be interpreted as independent or causal effects of parenting style. Longitudinal, multicentre studies with larger and more balanced samples and with measurement of dietary and socioeconomic factors are recommended to confirm these associations.

### 4.2. Clinical Implications and Future Directions

Two implications follow for clinical practice, both of which should be regarded as provisional given the exploratory nature of these findings. First, the finding that a substantial proportion of the association between the authoritative dimension and lower caries experience was transmitted through dental anxiety identifies anxiety as a potentially modifiable point of intervention. Unlike parenting style, dental anxiety can be addressed directly in the dental setting through established non-pharmacological behaviour-management approaches, and systematic reviews support the effectiveness of such interventions in children [9,10]. Routine screening for dental anxiety at the first pediatric visit, using a brief validated instrument, would allow these techniques to be applied selectively to children most likely to benefit. For the permissive dimension, the persistence of a direct association after adjustment for dental anxiety raises the possibility that behavioral pathways, including parental supervision of toothbrushing, dietary practices, and preventive attendance, may also contribute. These pathways were not measured in the present study and should therefore be examined directly in future research before specific parenting-style-based preventive strategies are recommended.

Several directions for further research follow from the limitations of this study. Longitudinal designs with repeated measurement of parenting, anxiety, and caries experience would allow the temporal ordering assumed by the mediation framework to be tested rather than assumed and would help to disentangle the reciprocal relationship between anxiety and disease experience. Studies that recruit purposively to achieve comparable numbers across parenting-style categories would permit the indirect pathway to be estimated with adequate precision for the authoritarian and permissive dimensions, which the present sample could not support. Measurement of dietary intake, socioeconomic position, parental education, and oral-hygiene behaviour would allow the behavioural route to be modelled explicitly rather than inferred and would establish whether the association between parenting style and caries experience persists after adjustment, a question on which the existing literature remains divided [19,32]. Finally, formal cross-cultural adaptation and validation of a parenting-style instrument for use in Arabic-speaking and multilingual Gulf populations would remove a source of uncertainty that applies not only to the present study but to the wider regional literature.

## 5. Conclusions

In this clinic-based sample, dental anxiety, dental caries experience, and parenting style were associated. Children classified in the small permissive subgroup had higher dental anxiety and greater caries experience than those classified as authoritative; however, these categorical findings should be interpreted cautiously because of the marked imbalance in subgroup sizes. In a multivariable mediation model, dental anxiety accounted for approximately 39% of the association between the authoritative dimension and caries experience. For the permissive dimension, no statistically significant indirect effect through dental anxiety was detected, while the direct association with caries experience remained significant, suggesting that other behavioural or family-related pathways may contribute. These findings should be regarded as exploratory: the parenting-style classification was heavily skewed towards the authoritative style, the authoritarian and permissive subgroups were small, dietary and socioeconomic confounders were not measured, and the cross-sectional design does not permit causal or temporal interpretation of these pathways. Within these constraints, the findings emphasize family-centred and psychologically informed approaches in pediatric oral healthcare, in which early recognition and management of dental anxiety are combined with parental guidance.

## Figures and Tables

**Figure 1:**
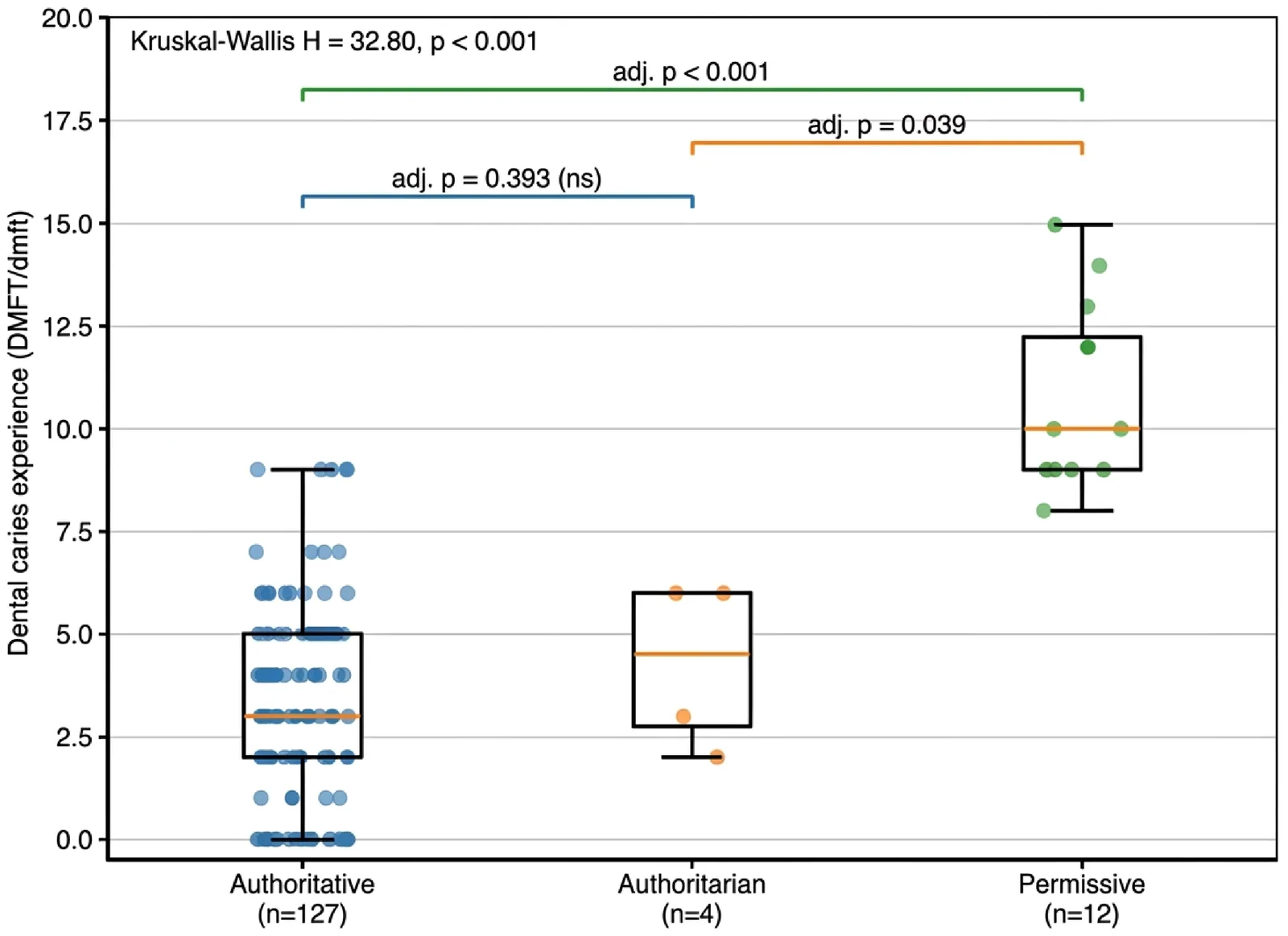
Comparison of dental caries experience across parenting styles. Boxplots represent the median and interquartile range, with whiskers extending to 1.5 × IQR. Pairwise comparisons were performed using Dunn’s post hoc test with Benjamini–Hochberg adjustment; adjusted *p*-values are shown.

**Figure 2:**
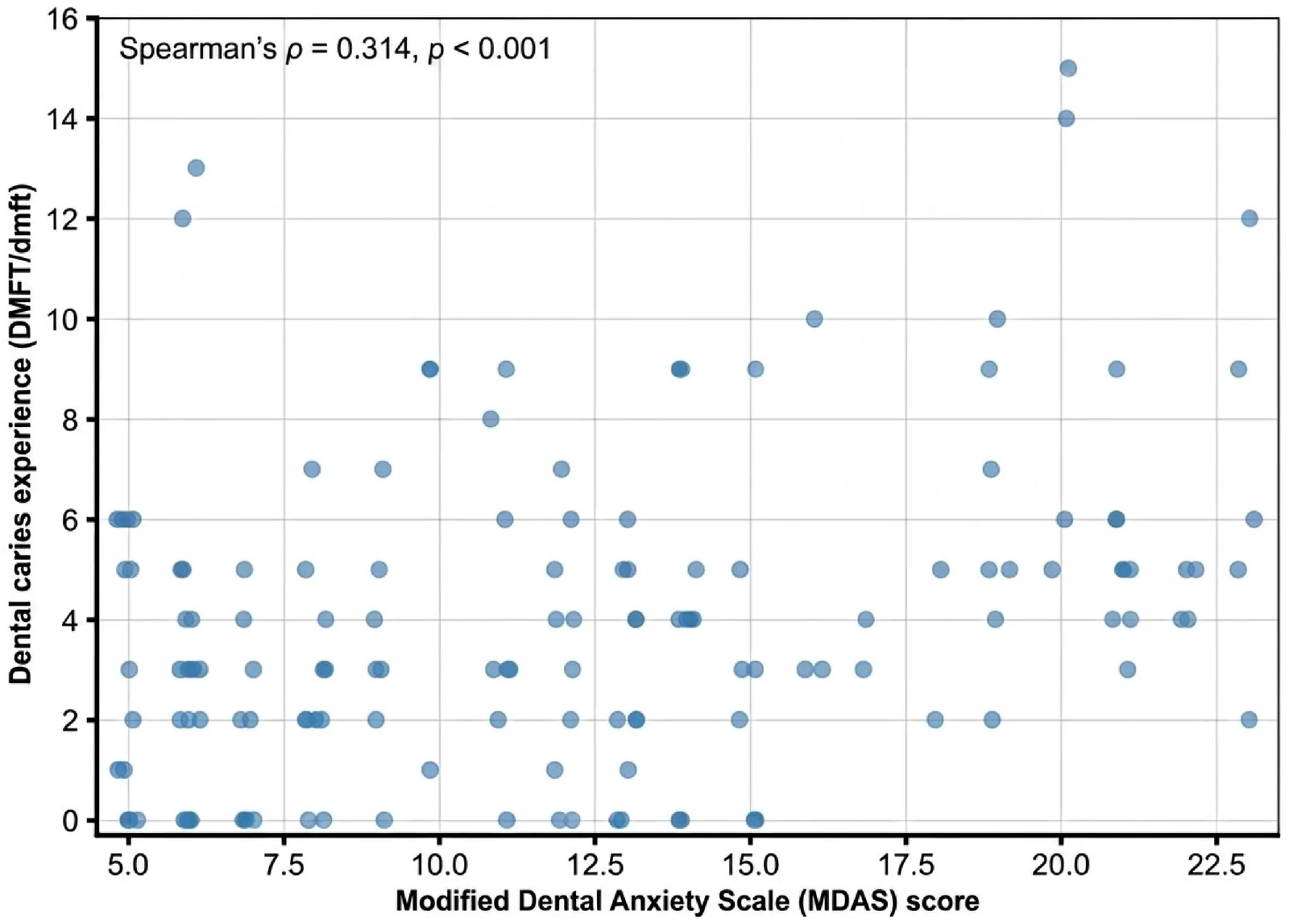
Correlation between dental anxiety and dental caries experience. Scatter plot showing the relationship between Modified Dental Anxiety Scale (MDAS) scores and DMFT/dmft scores. Horizontal jitter was applied to improve visualization of overlapping observations. A statistically significant positive correlation was observed (Spearman’s ρ = 0.314, *p* < 0.001).

**Table 1:** Participant characteristics, PSDQ dimension scores, and distribution of parenting styles and dental anxiety levels (*n* = 143).

Variable	Value
Age, years—mean ± SD (range)	8.92 ± 1.70 (6–12)
6–8 years—*n* (%)	59 (41.3)
9–12 years—*n* (%)	84 (58.7)
Sex—male/female, *n* (%)	71 (49.7)/72 (50.3)
DMFT/dmft—mean ± SD	3.94 ± 3.11
DMFT/dmft—median (Q1–Q3)	4 (2–5)
DMFT/dmft > 0—*n* (%)	119 (83.2)
MDAS score—mean ± SD	12.14 ± 5.56
MDAS score—median (Q1–Q3)	12 (8–16)
PSDQ authoritative—mean ± SD (median, Q1–Q3)	5.15 ± 1.08 (5.50, 5.00–5.90)
PSDQ authoritarian—mean ± SD (median, Q1–Q3)	2.37 ± 0.75 (2.40, 1.90–2.90)
PSDQ permissive—mean ± SD (median, Q1–Q3)	2.54 ± 0.96 (2.20, 2.00–2.90)
Classification margin—median (Q1–Q3); minimum	3.00 (2.60–3.30); 1.90
Parenting style—authoritative, *n* (%)	127 (88.8)
Parenting style—authoritarian, *n* (%)	4 (2.8)
Parenting style—permissive, *n* (%)	12 (8.4)
Dental anxiety—low (5–9), *n* (%)	58 (40.6)
Dental anxiety—moderate (10–18), *n* (%)	56 (39.2)
Dental anxiety—high (19–25), *n* (%)	29 (20.3)

PSDQ dimension scores are expressed on the six-point response metric used in this study (range 1–6). The classification margin is the difference between each parent’s highest and second-highest dimension score.

**Table 2:** Association between parenting style and dental anxiety.

Parenting Style	Low Anxiety	Moderate Anxiety	High Anxiety	*p*-Value
Authoritative (*n* = 127)	55 (43.3%)	51 (40.2%)	21 (16.5%)	0.016
Authoritarian (*n* = 4)	1 (25.0%)	2 (50.0%)	1 (25.0%)	
Permissive (*n* = 12)	2 (16.7%)	3 (25.0%)	7 (58.3%)	

Fisher’s exact test.

**Table 3:** Comparison of dental caries experience across parenting styles.

Parenting Style	*n*	Mean DMFT/dmft ± SD	Median (Q1–Q3)	*p*-Value
Authoritative	127	3.28 ± 2.32	3 (2–5)	<0.001
Authoritarian	4	4.25 ± 2.06	4.5 (2.75–6)	
Permissive	12	10.83 ± 2.29	10 (9–12.25)	

Kruskal–Wallis: H = 32.80, *p* < 0.001. Post hoc comparisons: Dunn’s test with Benjamini–Hochberg correction.

**Table 4:** Mediating effect of dental anxiety on the association between parenting-style dimensions and dental caries experience: multivariable model adjusted for the remaining two dimensions, age, and sex.

Dimension	Effect	Estimate	95% CI	*p*-Value
Authoritative	ACME	−0.260	−0.526 to −0.072	0.006
	ADE	−0.407	−1.084 to 0.184	0.183
	Total effect	−0.667	−1.368 to −0.057	0.032
Authoritarian	ACME	−0.130	−0.362 to 0.070	0.202
	ADE	0.088	−0.441 to 0.713	0.691
	Total effect	−0.042	−0.615 to 0.644	0.943
Permissive	ACME	−0.122	−0.344 to 0.066	0.192
	ADE	1.497	0.776 to 2.139	<0.001
	Total effect	1.376	0.605 to 2.049	<0.001

ACME, average causal mediation effect; ADE, average direct effect; CI, confidence interval. Estimates and 95% CIs were obtained using nonparametric bootstrapping with 5000 simulations. A non-significant indirect effect indicates that no statistically significant mediation was detected and does not establish that mediation is absent.

**Table 5:** Sensitivity analyses: average causal mediation effect (ACME) for each parenting-style dimension under alternative model specifications.

Specification	Authoritative	Authoritarian	Permissive
Separate single-dimension models, unadjusted	−0.153 (*p* = 0.007)	−0.019 (*p* = 0.823)	0.109 (*p* = 0.192)
Joint model, scores rescaled to five-point metric	−0.325 (*p* = 0.003)	−0.163 (*p* = 0.215)	−0.152 (*p* = 0.213)
Joint model, children aged 9–12 years only (*n* = 84)	−0.284 (*p* = 0.016)	−0.234 (*p* = 0.043)	−0.201 (*p* = 0.026)

All joint models are adjusted for the remaining two dimensions, age, and sex. The primary model is presented in Table 4. The rescaled model is shown for comparability with published values; because the rescaling is linear, it cannot alter inference.

## Data Availability

The data presented in this study are available on request from the corresponding authors. The data are not publicly available because they contain information that could compromise the privacy of the child participants.

## Abbreviations

The following abbreviations are used in this manuscript:

ACME	Average causal mediation effect
ADE	Average direct effect
CI	Confidence interval
DMFT/dmft	Decayed, missing, and filled teeth (permanent/primary dentition)
IQR	Interquartile range
MDAS	Modified Dental Anxiety Scale
PSDQ	Parenting Styles and Dimensions Questionnaire
SD	Standard deviation
STROBE	Strengthening the Reporting of Observational Studies in Epidemiology
WHO	World Health Organization
VIF	Variance inflation factor
